# Critical Elements of an Mpox Vaccination Model at the Largest Public Health Hospital System in the United States

**DOI:** 10.3390/vaccines11071138

**Published:** 2023-06-23

**Authors:** Anthony J. Lo Piccolo, Justin Chan, Gabriel M. Cohen, Ofole Mgbako, Robert A. Pitts, Radu Postelnicu, Andrew Wallach, Vikramjit Mukherjee

**Affiliations:** 1 Division of Infectious Diseases & Immunology, NYU Langone Health, New York, NY 10016, USA; 2 NYC Health + Hospitals (NYC H+H)/Bellevue, New York, NY 10016, USA; 3 Department of Medicine, NYU Grossman School of Medicine, New York, NY 10016, USA; 4 Department of Population Health, NYU Grossman School of Medicine, New York, NY 10016, USA

**Keywords:** mpox, mpox vaccine, JYNNEOS, vaccine equity, vaccine uptake, community outreach, vaccine rollout, public health emergency, emergency preparedness, special pathogen preparedness

## Abstract

In the spring of 2022, mpox spread to non-endemic countries, including the United States. In New York City (NYC), vaccine demand grew as quickly as case counts. With the leadership of the Regional Emerging Special Pathogens Treatment Center (RESPTC) at NYC Health and Hospitals/Bellevue (NYC H+H)—part of the largest public hospital system in the United States—an innovative vaccination model was established that overcame challenges involving health inequities, inadequate access, and lack of vaccine uptake, to successfully administer JYNNEOS vaccines to over 12,000 patients. Transmission has slowed since its peak in August 2022, which has been attributed to successful vaccination campaigns, infection-induced immunity, and behavioral changes among those at highest risk; however, a Centers for Disease Control and Prevention (CDC) assessment released on 4 April 2023 suggests jurisdictions with low vaccination levels (<35%) remain at risk for an mpox resurgence. Here, we summarize the critical aspects of our mpox vaccination model in NYC, which include integration into routine clinical care, prioritization of health equity, and reutilization of COVID-19 vaccination systems, to provide valuable insights for healthcare institutions as we move into the next stage of this ongoing outbreak.

## 1. Introduction

As of 26 April 2023, the United States has identified 30,361 cases of mpox [1], many of which occurred in men who have sex with men (MSM). Mirroring the COVID-19 outbreak, NYC was initially the epicenter of the 2022 mpox outbreak in the United States [2]. Unlike COVID-19, medical countermeasures against mpox were already at the disposal of medical professionals. However, global supply issues and bureaucratic challenges to maintain sufficient mpox vaccine in the US Strategic National Stockpile caused severe supply shortages early in the outbreak and forced local policymakers to make difficult decisions on who received the first vaccines. This was also a global health issue, as even the United States, the most resourced setting in the world, had difficulty obtaining adequate mpox vaccine supplies.

To best prioritize vaccines, JYNNEOS supplies were initially only offered as post-exposure prophylaxis (PEP) to those with known high-risk exposure to an individual with mpox [3,4]. By 3 June 2022, persons with occupational risk for exposure, including research laboratory personnel working with orthopoxviruses, clinical laboratory personnel performing diagnostic testing for orthopoxviruses, and healthcare worker (HCW) response teams who cared for patients infected with orthopoxvirus, became eligible for mpox vaccination [5]. Soon after, based on local epidemiology, the NYC Department of Health and Mental Hygiene (DOHMH) expanded vaccine eligibility to include MSM and/or transgender, gender non-conforming, or gender-nonbinary individuals aged 18 years or older who reported having multiple or anonymous sexual partners in the last 14 days but without a confirmed exposure (known as expanded post-exposure prophylaxis: PEP++) [3,4]. Pre-exposure prophylaxis (PrEP) is now available for at-risk groups who may not have multiple or anonymous sex partners, and people with HIV infection or other causes of immunosuppression that may anticipate mpox exposure [4].

While individuals aged 55 and above may have been vaccinated with the smallpox vaccination before smallpox was eradicated in the United States in 1972, the level to which vaccine immunity has waned over time remains uncertain [6,7,8]. Moreover, many of NYC H+H’s older or foreign-born patients may not have their smallpox vaccination status in their electronic medical records. For those reasons, prior smallpox vaccination status did not impact mpox vaccine eligibility [8].

A CDC report released on 4 April 2023 suggested jurisdictions with low mpox vaccination coverage, defined as <35% of PrEP-eligible individuals, were at risk of an mpox resurgence [9]. Many jurisdictions still have low mpox vaccination coverage, underscoring the importance of vaccines as an infectious disease mitigation tool. As the largest public health system in the United States, NYC H+H has always played a significant role in treating underserved communities and historically marginalized populations [10]. As such, NYC H+H’s involvement in mpox vaccinations ensured equity in distribution. The NYC H+H system has vaccinated 12,795 patients (Table 1), contributing to the 89% of eligible New Yorkers who received at least one mpox vaccine dose [11]. The success of the vaccination model enacted at NYC H+H, despite vaccine supply shortages, staffing hurdles, and early inequitable distribution due to processes put forth by regulatory agencies, can be attributed to close coordination with the DOHMH, just-in-time training (JITT) for staff, effective public education and outreach, prioritization of highest-risk individuals, increased accessibility, and strategically positioned vaccines.

## 2. Leveraging Pre-Existing COVID-19 Infrastructure to Step Up Mpox Vaccination

Fortuitously, the same leadership at NYC H+H and the DOHMH who had previously collaborated on the city’s response to COVID-19 were, again, working together to quell the mpox outbreak. The well-established DOHMH contact tracing unit was able to rapidly identify exposed persons eligible for PEP and link, via existing referral pathways, to the NYC H+H’s clinical facilities for PEP administration. Over 200 direct DOHMH referrals received subcutaneous PEP at Bellevue Hospital, including the first outpatient PEP vaccination in the city.

The experience of rolling out COVID-19 vaccinations gave leadership an understanding of the system’s infrastructure capabilities to respond to the mpox outbreak. Leaning heavily on the framework developed to address the COVID-19 pandemic, NYC H+H enacted an mpox-specific response spearheaded by the RESPTC, Bellevue Hospital. For example, Bellevue repurposed two vaccine hubs (outside of the clinics) in the COVID-19 vaccination center to become mpox vaccine hubs for PEP patients. The mpox station was staffed with one nurse trained in both JYNNEOS vaccine administration and identification of active skin lesions consistent with mpox, administrative staff to support scheduling and registration, and an on-call physician available for clinical support to the nurses and to answer questions on how to manage adverse reactions following vaccination.

Since patients eligible for PEP were at higher risk for developing mpox, these two stations were strategically placed adjacent to the exterior exits to minimize their presence in the health care facility, their interaction with others, and, thus, the possibility of transmission should the patient have developed active disease since the initial contact tracing. Of note, to further minimize this risk, patients scheduled to receive PEP were called the day prior and the morning of their appointment to ask for evidence of the development of active disease. Those patients scheduled for PEP vaccination who developed symptoms were not vaccinated, since vaccine efficacy is unknown after active infection [4]. Instead, they were brought in for testing. Because mpox can present similarly to other infectious agents affecting the skin, these patients were not only tested for mpox but herpes simplex virus, varicella virus, and an STI workup that included gonorrhea, chlamydia, syphilis, and HIV (with patient consent). Additionally, due to vaccine supply shortages and evidence for infection-induced immunity [12,13], patients who recovered from mpox were initially not vaccinated.

Having recently orchestrated a large-scale COVID-19 vaccine rollout, the hospital pharmacy was practiced in protocols for acquiring, storing, and distributing vaccines to the vaccine hub. The procedure for requesting vaccines from the hospital pharmacy was familiar to providers and nurses as well. Transmission-based PPE (e.g., an N95 mask, face shield, gown, and extended cuff examination gloves) used during the COVID-19 pandemic was also used by staff testing for mpox and administering mpox vaccines. JITT was sufficient to refresh staff as to the PPE requirements. The importance and familiarity with PPE were understood by staff because of the education and usage throughout the COVID-19 pandemic.

## 3. Ramping Up Vaccine Administration to Meet Expanding Eligibility

**Figure 1:**
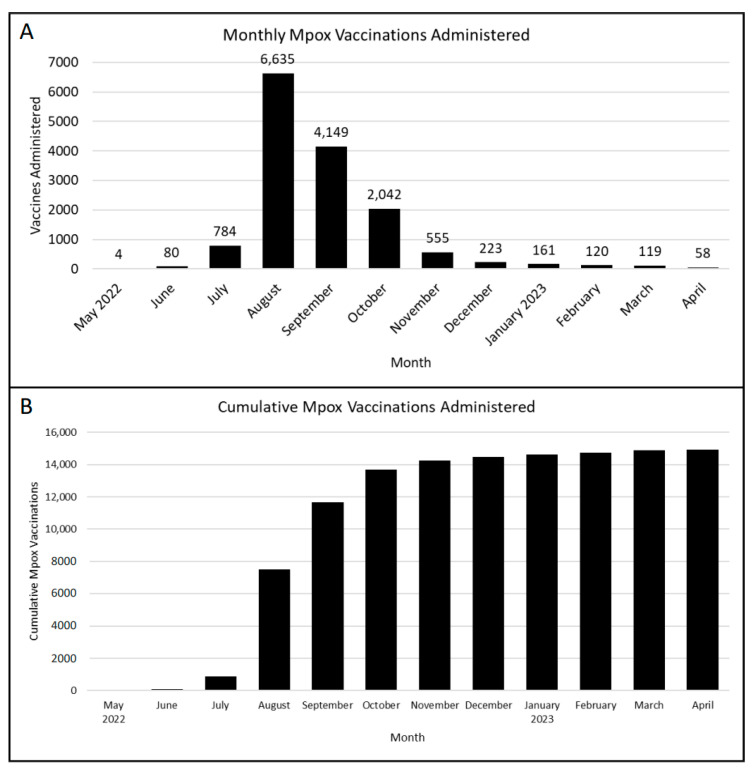
(**A**) The number of mpox vaccine doses administered at NYC H+H sites from 1 May 2022 through 26 April 2023. NYC H+H quickly stood up its vaccination capabilities, as evidenced by an 8-fold increase in doses administered from July 2022 to August 2022. (**B**) Cumulative number of mpox doses administered at NYC H+H site. Over 14,000 mpox vaccine doses were administered to NYC H+H patients across NYC.

**Figure 2:**
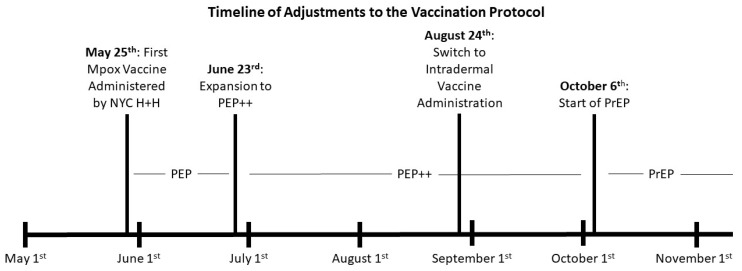
Timeline of significant adjustments to the vaccination protocol, May 2022—November 2022. The first mpox vaccine administered by NYC H+H occurred on 25 May 2022. Since then, the eligibility was expanded to PEP++ on 23 June 2022, and then PrEP on 6 October 2022. The switch to intradermal vaccine administration occurred on 24 August 2022.

NYC H+H was able to ramp up to a sustainable vaccination program, as evidenced by our daily and cumulative vaccination curves (Figure 1). Due to improvements in vaccine supply, eligibility was extended to include PEP++ in June 2022 (Figure 2). PEP++ was administered at three vaccination centers opened by the DOHMH at sexual health clinics; subsequently, NYC H+H added three additional mpox vaccine sites working in collaboration with the DOHMH. Educational materials were developed to train providers how to identify eligible patients, order vaccines in the electric health records, and address basic mpox clinical questions. Registered nurses (RNs) were also trained to identify eligible patients, in addition to training about vaccine administration, consenting procedures, and managing the clinic’s vaccine supply.

To increase vaccine stock, the CDC switched to intradermal administration over subcutaneous, since less vaccine product is required [14,15,16]. The switch to intradermal vaccination in late August 2022 helped stabilize NYC’s vaccine supply and allowed for nearly a five-fold increase in vaccine appointments [17]. There are limited data on the effectiveness and reactogenicity of intradermal over subcutaneous administration [18,19] and concerns about site-associated hyperpigmentation and scarring associated with intradermal vaccination [15]. As such, patients with a known history of keloids continued to receive the vaccine by the subcutaneous route. Now that the vaccine supply has stabilized, most jurisdictions (including NYC) have reverted to subcutaneous administration.

By early October 2022, the vaccine supply had adequately increased to allow for expansion to PrEP [20]. The PrEP model also brought about a shift in the mpox vaccination strategy. Local hospitals and clinics became the primary locations to receive mpox vaccines, in place of vaccination centers. At this stage, NYC H+H utilized their Pride Health Centers (dedicated to the provision of gender-affirming care to the LGBTQ+ community) and the Virology/Infectious Disease clinics (dedicated to treating people with HIV) to administer vaccines Mondays through Saturdays. The Pride Health Center at NYC H+H/Bellevue Hospital alone provides care to over 300 underserved and culturally diverse LGBTQ+ New Yorkers [21]. Patients who received their mpox vaccine at the Pride Health Center were also screened for STIs—gonorrhea, chlamydia, syphilis, and HIV—during their vaccination visit as part of the standard of care. Patients regularly seen at Bellevue Hospital’s Pride Health Center or in primary care received their mpox vaccines in the Pride Health Center, while patients regularly seen in the Virology clinic, or any outside referrals, received their vaccine in the Virology clinic. Incorporating the Virology clinic was strategic, as HIV and mpox are syndemic. Recent studies have found around 40% of those diagnosed with mpox also had HIV [22,23]. NYC H+H continues to accommodate vaccine appointments daily at all seven Pride Health Centers and all Virology clinics throughout the system.

Due to uncertainty around virus virulence and severity, as well as unfamiliarity with the mpox vaccine early on in the outbreak, obtaining staff buy-in was difficult. Providers added treating, testing, and vaccinating mpox patients to their already busy daily schedules. Staffing issues were addressed by educating and in-servicing RNs and providers, as well as utilizing agency staffing to expand staffing capacity. Bellevue Hospital’s Pride Health Center trained 8 RNs and educated 30 primary care providers, while Bellevue’s Virology clinic trained 3 RNs and educated 12 primary care providers.

Providers played an important role in the Pride Health Centers and Virology clinics to oversee vaccine administration. They were the contact persons for patient questions and concerns, ordered vaccine doses in the electronic health record (EPIC), and were present to handle any clinical issues that arose. Providers also provided active outreach by identifying and contacting eligible patients to inform them of their eligibility and upcoming available vaccine appointments. RNs’ vital role in administering mpox vaccines, attending to patients, and monitoring the Pride Health Center and Virology clinic’s vaccine stock cannot be understated.

## 4. Incorporating Vaccine Equity

Attaining equitable access was difficult, particularly at the onset of the outbreak. Quickly rolling out a large-scale vaccination model led to inevitable operational difficulties. With so few vaccine appointments available, the DOHMH centralized vaccine scheduling for all vaccination centers via an online scheduling platform. Unfortunately, only those with better internet access and the time to monitor the scheduling website for new appointments received the first vaccines. Also, initial appointments were often during work hours, making it difficult for those who could not get off work to make an appointment.

To address mpox vaccine scheduling inequities, NYC H+H leaders advocated for a telephone hotline to accompany the online scheduling platform. Recognizing the necessity, DOHMH established a hotline that opened accessibility for those without internet access. Significantly, NYC H+H and other organizations caring for high-priority at-risk populations received an allotment of daily appointment slots at PEP++ vaccination centers for their patients, many of whom were racial and ethnic minorities disproportionately affected by the outbreak. The NYC H+H team then conducted active outreach to its at-risk patients and scheduled their vaccine appointments. Thus, the carve-out of those slots ensured greater vaccine equity.

For some patients, the stigma associated with mpox diagnosis may have been a hindrance to seeking testing and vaccination. Tackling this challenge required effective community outreach efforts. NYC H+H’s mobile vaccine units were deployed midway through the outbreak to LGBTQ+ venues to provide vaccines to the communities that needed them most. The mobile units were staffed by NYC H+H vendors, previously utilized for COVID-19-related efforts. An mpox education campaign increased the community’s awareness of potential risks and helped change the behaviors of individuals without the need to close down venues or events to stop the spread.

Another community outreach strategy was the delivery of vaccines to LGBTQ+ community clinics and organizations as quickly as possible. Positioning our vaccines at Pride Health Centers and Virology clinics allowed for those most at risk to have easy access to the vaccine. Moreover, the patient–provider relationship was leveraged to spur vaccine awareness and uptake. This strategy allowed for open communication between providers and patients. Hospital providers and staff also helped patients schedule vaccine appointments and followed up with them to ensure they attended their appointments. Engaging the providers that MSM patients already see and trust was an effective strategy to increase vaccine awareness, uptake, accessibility, and equity. The success of this approach speaks to the patient–provider trust cultivated at NYC H+H. A fragile commodity, maintaining community trust was critical to the effectiveness of mpox vaccine outreach.

## 5. Outlook

NYC H+H has vaccinated over 12,000 patients since May 2022 (Figure 1). Thanks to the efforts of the staff in the NYC H+H system, as well as hospitals and community centers throughout the city, 99,079 New Yorkers have received at least one dose of the mpox vaccine as of 25 April 2023 [11]. This can be attributed, in part, to the infrastructure developed at NYC H+H. Through the preparatory efforts, maintained by the Special Pathogens Program at NYC H+H/Bellevue, the hospital system rapidly trained staff to inject vaccines in arms. This highlights the importance of maintaining hospital readiness to address emerging special pathogen outbreaks from their onset. These readiness capabilities include bringing multidisciplinary teams together to develop policies and procedures, continuing staff education and training, maintaining personal protective equipment supplies, identifying space for stand-up emergency services (such as vaccine clinics), and sustaining relationships with local health departments.

As of June 2023, mpox continues to spread in populations outside of Africa [24,25,26]. Although NYC has successfully vaccinated most of the at-highest-risk populations and immunologic studies suggest a degree of long-term immunological memory [13,27,28], it is unknown if or when vaccine-induced immunity or natural immunity wanes [29]. For this reason, it is critical to continue the surveillance of mpox cases, conduct vaccine effectiveness evaluations, and pursue studies to assess the immunogenicity of vaccination over time [30,31]. A large majority of the general population has not been vaccinated, and the possibility of spread amongst groups outside of MSM social networks persists.

Healthcare facilities must think carefully about the occupational risk to their HCWs. NYC H+H has administered some PEP to HCWs with mpox exposure through patient contact. We have a history of vaccinating HCWs at occupational risk of infectious disease exposure. The initial administration of the COVID-19 vaccine prioritized HCWs, due to their high risk of occupational exposure and the infectability of SARS-CoV-2. Since mpox is not commonly transmitted through a respiratory route and is more commonly transmitted only once signs/symptoms are present [32,33], its asymptomatic transmission is uncertain [34], and mpox vaccines at the time were in short supply, HCWs were not prioritized by the CDC in the initial round of vaccinations. Another reason for this decision was the effectiveness of PPE. If wearing recommended PPE when treating suspected or confirmed mpox patients, unvaccinated HCWs are at low risk of infection to mpox [35,36,37]. However, a majority of workplace infections have occurred due to breaches in PPE or in the absence of PPE use [38,39,40].

Difficulties in gathering epidemiological risk factors or the active disclosure of risk upon presentation to the healthcare system have led to HCW and patient exposures. For example, a patient admitted to Bellevue Hospital’s psychiatric unit went undiagnosed with mpox until day 4 of admission due to a lack of disclosure of risk. Because transmission-based PPE was not required on the psychiatric unit, 29 patients and 84 staff members were exposed, all of whom were monitored and, thankfully, never developed mpox. Thus, the risk of mpox infecting HCWs is still present. The CDC’s Morbidity and Mortality Weekly Report (MMWR) from 3 June 2022 included a recommendation for JYNNEOS PrEP vaccination for HCW response teams who care for patients infected with orthopoxvirus and clinical and research laboratory personnel working with orthopoxviruses [5]. Now that supply issues have stabilized and many of the at-risk populations are vaccinated, we should identify HCWs who, due to the nature of their work, are more susceptible to mpox exposure.

Regardless of vaccination rates, NYC is an international travel hub and is always at an elevated risk for an infectious disease outbreak. In the age of globalization, inadequacies to contain the spread of mpox in one region of the world impacts international hubs such as NYC, which see thousands of international travelers daily. Our global interconnectivity underscores the importance for healthcare institutions in the New York tri-state area and across the US to remain vigilant for the possibility of patients seeking mpox testing, treatment, and vaccines. Looking forward, we must ensure that we have an adequate vaccine supply to vaccinate all of the at-risk population. There is a high probability that we will see a resurgence in national infections with the current level of mpox vaccination coverage in the US [11]. We must continue to monitor this outbreak and, as it evolves, reevaluate at-risk populations and update vaccine eligibility to reflect those most at-risk. Ongoing preparedness efforts such as adding the mpox vaccine to the list of routine healthcare vaccines for those at increased risk, identifying best practices based on the lessons learned, and maintaining awareness and community outreach efforts are critical.

## 6. Conclusions

NYC H+H, led by the RESPTC at Bellevue Hospital, deployed a multidisciplinary team of providers, RNs, epidemiologists, and pharmacists to provide mpox vaccination services to eligible patients. A strong relationship with DOHMH leadership and interdepartmental collaboration was critical to coordinate the different facets of the mpox vaccine rollout. Identifying patients eligible for mpox vaccination, maintaining vaccine supply, ensuring adherence to infection prevention controls, scheduling initial and second vaccine appointments, and building equity-focused workflows into procedures were responsible for our cohesive and efficient vaccination model, along with interdisciplinary efforts. Additionally, the response to the COVID-19 pandemic better prepared the hospital system to handle this mpox outbreak. The relationships and infrastructure developed during the COVID-19 pandemic were the basis of our mpox response, including our vaccination model. NYC H+H built trust with the MSM/LGBTQ+ community to effectively reach the most at-risk individuals. The ability to vaccinate so many individuals across the city speaks to the success of NYC H+H’s innovative mpox vaccination model.

## Figures and Tables

**Table 1:** Demographics of mpox vaccination at NYC H+H.

NYC H+H Mpox Vaccine Demographics (N = 12,795)	Patients Who Received at Least One Dose of the JYNNEOS Vaccine	% of Total Administered ^§^
Age		
<18 years	28	0.22%
18–24 years	1008	7.88%
25–34 years	4625	36.15%
35–44 years	3307	25.84%
45–54 years	1742	13.61%
55–64 years	1520	11.88%
65–74 years	464	3.63%
75–84 years	95	0.74%
85+ years	6	0.05%
Gender		
Male	5852	45.74%
Female	543	4.24%
Gender Non-Binary/ Non-Conforming	240	1.88%
Transgender Male	46	0.36%
Transgender Female	90	0.70%
None of the Above/Unknown	6024	47.08%
Race *		
Asian	1027	8.03%
American Indian or Alaskan Native	42	0.33%
Black or African American	2327	18.19%
Native Hawaiian or Other Pacific Islander	30	0.23%
White	6030	47.13%
Other	2643	20.66%
Unknown	1033	8.07
Ethnicity		
Hispanic or Latino	2788	21.79%
Not Hispanic or Latino	9999	78.15%
Unknown	8	0.06%

NYC H+H opened access to underserved and historically marginalized communities in the rollout of the mpox vaccine. § Percentage calculated using total patients vaccinated, N = 12,795. * Some individuals reported multiple races.

## Data Availability

The data presented in this study are available on request from the corresponding author. The data are not publicly available as they are obtained using internal NYC H+H records. Data on NYC H+H mpox vaccinations were obtained using SlicerDicer within the NYC H+H system’s electronic medical record, EPIC. SlicerDicer was used to perform searches on large patient populations. No patient PHI was obtained to create Figure 1 or Table 1.

## References

[1] (2023). 2022 U.S. Map & Case Count _ Mpox _ Poxvirus _ CDC. https://www.cdc.gov/poxvirus/mpox/response/2022/us-map.html#print.

[2] (2023). Mpox 2022 Summary: Cases. https://www.nyc.gov/assets/doh/downloads/pdf/monkeypox/mpox-response-data-summary.pdf.

[3] (2023). Monkeypox (Mpox) Vaccination-NYC Health. https://www.nyc.gov/site/doh/health/health-topics/monkeypox-vaccination.page.

[4] (2023). Mpox Vaccination. https://www.cdc.gov/poxvirus/mpox/interim-considerations/overview.html#print.

[5] Rao A.K., Petersen B.W., Whitehill F., Razeq J.H., Isaacs S.N., Merchlinsky M.J., Campos-Outcalt D., Morgan R.L., Damon I., Sánchez P.J. (2021). Use of JYNNEOS (Smallpox and Monkeypox Vaccine, Live, Nonreplicating) for Preexposure Vaccination of Persons at Risk for Occupational Exposure to Orthopoxviruses: Recommendations of the Advisory Committee on Immunization Practices-United States, 2022. Morb. Mortal. Wkly. Rep..

[6] Rimoin A.W., Mulembakani P.M., Johnston S.C., Smith J.O.L., Kisalu N.K., Kinkela T.L., Blumberg S., Thomassen H.A., Pike B.L., Fair J.N. (2010). Major increase in human monkeypox incidence 30 years after smallpox vaccination campaigns cease in the Democratic Republic of Congo. Proc. Natl. Acad. Sci..

[7] Karem K.L., Reynolds M., Hughes C., Braden Z., Nigam P., Crotty S., Glidewell J., Ahmed R., Amara R., Damon I.K. (2007). Monkeypox-induced immunity and failure of childhood smallpox vaccination to provide complete protection. Clin. Vaccine Immunol..

[8] (2023). JYNNEOS Vaccine for Mpox: Frequently Asked Questions. https://www.nyc.gov/assets/doh/downloads/pdf/monkeypox/jynneos-vaccine-faq.pdf#:~:text=Should%20people%20who%20previously%20received,vaccine%20may%20lessen%20over%20time.

[9] (2023). Risk Assessment of Mpox Resurgence and Vaccination Considerations. medRxiv.

[10] (2022). NYC Health + Hospitals Patients. https://equity.nyc.gov/city-services/nyc-health-hospitals-patients.

[11] (2023). Mpox Vaccine Coverage by Jurisdiction for Population at Risk for Mpox. https://www.cdc.gov/poxvirus/mpox/cases-data/mpx-jynneos-vaccine-coverage.html#print.

[12] Hammarlund E., Lewis M.W., Hanifin J.M., Mori M., Koudelka C.W., Slifka M.K. (2010). Antiviral Immunity following Smallpox Virus Infection: A Case-Control Study. J. Virol..

[13] Sivapalasingam S., Kennedy J.S., Borkowsky W., Valentines F., Zhan M., Pazoles P., Paolino A., Ennis F.A., Steigbigel N.H. (2007). Immunological Memory after Exposure to Variola Virus, Monkeypox Virus, and Vaccinia Virus. J. Infect. Dis..

[14] Wilck M.B., Seaman M.S., Baden L.R., Walsh S.R., Grandpre L.E., Devoy C., Giri A., Kleinjan J.A., Noble L.C., Stevenson K.E. (2010). Safety and Immunogenicity of Modified Vaccinia Ankara (ACAM3000): Effect of Dose and Route of Administration. J. Infect. Dis..

[15] Frey S.E., Wald A., Edupuganti S., Jackson L.A., Stapleton J.T., El Sahly H., El-Kamary S.S., Edwards K., Keyserling H., Winokur P. (2015). Comparison of lyophilized versus liquid modified vaccinia Ankara (MVA) formulations and subcutaneous versus intradermal routes of administration in healthy vaccinia-naïve subjects. Vaccines.

[16] Brooks J., Marks P., Goldstein R., Walensky R. (2022). Intradermal Vaccination for Monkeypox—Benefits for Individual and Public Health. N. Engl. J. Med..

[17] (2022). City to Adopt New Intradermal Monkeypox Vaccination Strategy to Reach More New Yorkers—NYC Health. https://www.nyc.gov/site/doh/about/press/pr2022/new-intradermal-monkeypox-vaccination-strategy-to-reach-more-newyorkers.page.

[18] Frey E.S., Stapleton J.T., Ballas Z.K., Rasmussen W.L., Kaufman T.M., Blevins T.P., Jensen T.L., Davies D.H., Tary-Lehmann M., Chaplin P. (2021). Human Antibody Responses Following Vaccinia Immunization Using Protein Microarrays and Correlation with Cell-Mediated Immunity and Antibody-Dependent Cellular Cytotoxicity Responses. J. Infect. Dis..

[19] Johansen P., Mohanan D., Martínez-Gómez J.M., Kündig T.M., Gander B. (2010). Lympho-geographical concepts in vaccine delivery. J. Control. Release.

[20] (2022). City Expands Eligibility for Monkeypox (MPV) Vaccination and Opens More Than 30,000 New Appointments. https://www.nyc.gov/site/doh/about/press/pr2022/city-expands-mpv-vaccine-eligibility.page.

[21] The Pride Health Center at NYC Health + Hospitals/Bellevue NYC Health + Hospitals. https://www.nychealthandhospitals.org/bellevue/services/pride-health-center/.

[22] Curran K.G., Eberly K., Russell O.O., Snyder R.E., Phillips E.K., Tang E.C., Peters P.J., Sanchez M.A., Hsu L., Cohen S.E. (2022). HIV and Sexually Transmitted Infections Among Persons with Monkeypox—Eight, U.S. Jurisdictions, 17 May–22 July 2022. Morb. Mortal. Wkly. Rep..

[23] Philpott D., Hughes C.M., Alroy K.A., Kerins J.L., Pavlick J., Asbel L., Crawley A., Newman A.P., Spencer H., Feldpausch A. (2022). Epidemiologic and Clinical Characteristics of Monkeypox Cases-United States, 17 May–22 July 2022. Morb. Mortal. Wkly. Rep..

[24] (2023). *Health Alert Network (HAN)—00490_Potential Risk for New Mpox Cases*; American Association for the Advancement of Science: Washington, DC, USA. https://emergency.cdc.gov/han/2023/han00490.asp#print.

[25] Ward T., Christie R., Paton R.S., Cumming F., Overton C.E. (2022). Transmission dynamics of monkeypox in the United Kingdom: Contact tracing study. BMJ.

[26] Miura F., van Ewijk C.E., Backer J.A., Xiridou M., Franz E., de Coul E.O., Brandwagt D., van Cleef B., van Rijckevorsel G., Swaan C. (2022). Estimated incubation period for monkeypox cases confirmed in the Netherlands, May 2022. Eurosurveillance.

[27] Priyamvada L., Carson W.C., Ortega E., Navarra T., Tran S., Smith T.G., Pukuta E., Muyamuna E., Kabamba J., Nguete B.U. (2022). Serological responses to the MVA-based JYNNEOS monkeypox vaccine in a cohort of participants from the Democratic Republic of Congo. Vaccine.

[28] Chan C.E.Z., Wong S.K.K., Yazid N.B.M., Ng O.T., Marimuthu K., Chan M., Howe H.S., Leo Y.-S., Leung B.P., Vasoo S.S. (2022). Residual Humoral Immunity Sustained Over Decades in a Cohort of Vaccinia-Vaccinated Individuals. J. Infect. Dis..

[29] (2022). JYNNEOS Vaccine-CDC. https://www.cdc.gov/poxvirus/mpox/interim-considerations/jynneos-vaccine.html#:~:text=Peak.

[30] Thy M., Peiffer-Smadja N., Mailhe M., Kramer L., Ferré V.M., Houhou N., Tarhini H., Bertin C., Beaumont A.-L., Garé M. (2022). Breakthrough Infections after Postexposure Vaccination against Mpox. N. Engl. J. Med..

[31] (2023). Monkeypox: Update in France as of 23 March 2023. https://www.santepubliquefrance.fr/les-actualites/2023/variole-du-singe-point-de-situation-en-france-au-23-mars-2023.

[32] How It Spreads_Mpox_CDC. Centers for Disease Control and Prevention. Published 2 February 2023. https://www.cdc.gov/poxvirus/mpox/if-sick/transmission.html.

[33] Beeson A., Styczynski A., Hutson C.L., Whitehill F., Angelo K.M., Minhaj F.S., Morgan C., Ciampaglio K., Reynolds M.G., McCollum A.M. (2023). Mpox respiratory transmission the state of the evidence. Lancet.

[34] (2023). Science Brief: Detection and Transmission of Mpox(Formerly Monkeypox) Virus During the 2022 Clade IIb Outbreak. https://www.cdc.gov/poxvirus/mpox/about/science-behind-transmission.html#:~:text=Incorporated.

[35] Fleischauer A.T., Kile J.C., Davidson M., Fischer M., Karem K.L., Teclaw R., Messersmith H., Pontones P., Beard B.A., Braden Z.H. (2005). Evaluation of Human-to-Human Transmission of Monkeypox from Infected Patients to Health Care Workers. Clin. Infect. Dis..

[36] Petersen B.W., Kabamba J., McCollum A.M., Lushima R.S., Wemakoy E.O., Tamfum J.-J.M., Nguete B., Hughes C.M., Monroe B.P., Reynolds M.G. (2018). Vaccinating against monkeypox in the Democratic Republic of the Congo. Antivir. Res..

[37] Marshall K.E., Barton M., Nichols J., de Perio M.A., Kuhar D.T., Spence-Davizon E., Barnes M., Herlihy R.K., Czaja C.A., Abbey T. (2022). Health Care Personnel Exposures to Subsequently Laboratory-Confirmed Monkeypox Patients—Colorado, 2022. MMWR Morb. Mortal. Wkly. Rep..

[38] Vaughan A., Aarons E., Astbury J., Brooks T., Chand M., Flegg P., Hardman A., Harper N., Jarvis R., Mawdsley S. (2020). Human-to-Human Transmission of Monkeypox Virus, United Kingdom, October 2018. Emerg. Infect. Dis..

[39] Szkiela M., Wiszniewska M., Lipińska-Ojrzanowska A. (2023). Monkeypox (Mpox) and Occupational Exposure. Int. J. Environ. Res. Public Health.

[40] Caldas J.P., Valdoleiros S.R., Rebelo S., Tavares M. (2022). Monkeypox after Occupational Needlestick Injury from Pustule. Emerg. Infect. Dis..

